# Lead exposure may affect gingival health in children

**DOI:** 10.1186/s12903-018-0547-x

**Published:** 2018-05-04

**Authors:** Borany Tort, Youn-Hee Choi, Eun-Kyong Kim, Yun-Sook Jung, Mina Ha, Keun-Bae Song, Young-Eun Lee

**Affiliations:** 1 Department of Preventive Dentistry, School of Dentistry, Kyungpook National University, 2177 Dalgubeol-daero, Jung-gu, Daegu, 700-412 Republic of Korea; 2 Department of Dental Hygiene, College of Science and Technology, Kyungpook National University, 386 Gajangdong, Sangju, 742-711 Republic of Korea; 3 Department of Preventive Medicine, Dankook University College of Medicine, 119, Dandae-ro, Dongnam-gu, Cheonan-si, Chungnam 330-714 Republic of Korea; 4 Department of Dental Hygiene, Daegu Health College, 15 Youngsong-Ro, Buk-Gu, Daegu, 702-722 Republic of Korea

**Keywords:** Gingivitis, Pathology, Oral hygiene

## Abstract

**Background:**

Several studies have reported the harmful effects of lead poisoning. However, the relationship between lead exposure and oral health of children has not been well defined. The aim of this study was to investigate the relationship between blood lead level (BLL) and oral health status of children.

**Methods:**

A total of 351 children (aged 7–15 years) were recruited from the pilot data of the Korean Environmental Health Survey in Children and Adolescents, which was designed to examine environmental exposure and children’s health status in South Korea. Blood samples were taken to determine BLLs and oral examinations were performed to assess oral health parameters, including community periodontal index (CPI), gingival index (GI), and plaque index (PI). Information regarding socioeconomic status, oral hygiene behavior, and dietary habits was collected from parents and guardians.

**Results:**

The participants were divided equally into four quartiles, with quartile I comprised of children with the lowest BLLs. There were significant differences for PI (*p* < 0.05) among the quartile groups. Using logistic regression models, we found a significant relationship between BLL and oral health parameters. The crude odds ratios for CPI, GI, and PI in the third quartile were 5.24 (95% CI: 1.48-18.56), 4.35 (95% CI: 1.36-13.9), and 4.17 (95% CI: 1.50-11.54), respectively, while the age and gender-adjusted odds ratios were 7.66 (95% CI: 1.84-31.91), 6.80 (95% CI: 1.80-25.68), and 3.41 (95% CI: 1.12-10.40), respectively. After adjustments for age, gender, parent education level, and frequency of tooth brushing, the adjusted odds ratios were 7.21 (95% CI: 1.72-30.19), 6.13 (95% CI: 1.62-23.19), and 3.37 (95% CI: 1.10-10.34), respectively.

**Conclusions:**

A high BLL might be associated with oral health problems in children, including plaque deposition and gingival diseases.

## Background

Lead is a heavy metal widely employed in automobiles, gasoline, lubricants, and decolorizing agents, including other applications such as agriculture as an insecticide [1]. In the medical field, lead is used to produce antifungal agents and provide radiation protection [2]. However, reports of symptoms of lead poisoning are common and have been known since at least the second century B.C, with such poisoning in lead industrial workers and children first reported in the nineteenth century [3].

The detrimental effects of lead poisoning are related to the amount of lead accumulated in the body and blood lead level (BLL) is considered to be associated with physical health, mental health, the immune system, and functional growth [4]. Also, lead exposure has been reported to have an impact on human intelligence and social behavior, with life-long adverse effects [5, 6].

In addition to these general health risks, lead exposure can have harmful effects on oral health. Such exposure can affect enamel formation leading to caries production, delayed dental enamel formation, worsened dental fluorosis, and periodontal bone loss, as well as increased tooth loss in men [7–14]. A study by Saraiva et al. found that BLL was significantly associated with periodontal health in adult participants [15]. Another study conducted with Thai children living in a shipyard industrial area showed that there was a significantly positive correlation between high BLL and periodontal problems, including the presence of deep pockets, particularly teeth 16 and 46 [16].

Children tend to absorb more lead into their bodies than adults because of a higher rate of metabolism as well as a physical tendency to inhale lead from polluted air. Moreover, child gastrointestinal organs seem to absorb lead more easily [17]. Although some studies have speculated regarding a possible relationship between BLL and dental caries in children [18, 19], few have assessed the association between BLL and periodontal status in child subjects. The aim of present study was to investigate the relationship between BLL and oral health status in Korean children.

## Methods

### Study population

A total of 351 children ranging from 7 to 15 years old living in two cities in South Korea (Incheon city and Cheonan city) were recruited from the participants of the pilot study of the Korean Environmental Health Survey in Children and Adolescents [20]. That study aimed to show the environmental exposure and health of Korean children who were sampled with a national representativeness by use of a questionnaire survey, clinical tests, and physical examinations in 2011 and 2012. Of 351 subjects, 137 agreed to participate, were undertook oral health examination, and included in this study. The present study was approved by the institutional review board of a university hospital (IRB No. DKUH 2012-10-003-001). Parents or guardians of the subjects provided written informed consent for their participation, and completed questionnaires regarding their socioeconomic status and the oral hygiene behaviors of their children.

### Measurement of blood lead levels

Whole blood (3–5 ml) was drawn from the subjects and sealed in a heparin containing tube. Lead levels were determined using atomic absorption spectrophotometry (800 Zeeman correction; Spectral AA, Varian, NSW, Australia) at a commercial laboratory. The coefficient of variation of the blood lead measurements was 4.9%.

### Oral health examinations

Oral health examinations were conducted by 1 dentist and 2 dental hygienists trained according to the World Health Organization (WHO) guidelines on oral health surveys [21]. The dentist performed oral examinations using a dental mirror and periodontal probe under artificial light with the subject in a portable dental chair in order to detect decayed, missing, and filled surfaces of permanent teeth (DMFT), and also determined community periodontal index (CPI), gingival index (GI), and plaque index (PI) values [21–24]. The CPI, GI, and PI values of teeth 11, 16, 26, 31, 36, and 46 were assessed in the present study. CPI was scored as follows: 0 (healthy), 1 (bleeding following probing), 2 (presence of dental calculus). Because the presence of dental calculus is not an indicator of periodontal health in children, 11 participants whose CPI scores of six representative teeth were only 0 and 2 were excluded in the analysis for CPI. Also participants whose one of CPI scores of six representative teeth was 1 regardless of the rest were classified as having CPI score of 1. Given that children scarcely have periodontitis and are likely to have pseudo-periodontal pockets due to tooth eruption, CPI scores of 3 and 4 were not used.

### Socioeconomic status and oral hygiene behavior

Information regarding potential covariates was obtained from questionnaires completed by parents and guardians. We surveyed socioeconomic variables including family income, parent education level, father and mother’s occupations, oral hygiene behaviors such as frequency of tooth cleaning and frequency of intake of sugar-containing sweets, dental treatment demands, and history of decayed teeth.

### Statistical analysis

The subjects were divided into 4 equal quartiles based on measured BLL, with quartile I comprised of children with the lowest BLLs. Analysis of variance (ANOVA) and a Chi-square test were used to compare the covariates and oral health parameters among the quartiles. One crude and two adjusted logistic regression models were used to explore the relationship between BLL and oral health parameters. Two adjusted odds ratios (OR) were calculated.

The first model adjusted for age and gender, and the other added parent education level, and frequency of tooth brushing additionally as confounders. SPSS (ver. 20.0, IBM, NY, USA) was utilized for the analyses, with statistical significance considered at *p* < 0.05.

## Results

Table 1 shows the average measured BLLs according to demographic characteristics, socioeconomic status, oral hygiene behavior, dietary habits, and clinical oral parameters. The mean age of the subjects was 10.9 ± 2.1 years and 69 were girls (50.4%). Overall mean BLL was 1.25 ± 0.43 μg/dl, ranging from 0.36 to 2.90 μg/dl. There were no statistically significant differences observed among those factors.

**Table 1:** Mean blood lead level according to socioeconomic status and oral hygiene behavior in school-age children

	N	Pb (μg/dl) Mean ± SD
Total participants	137	1.25 ± 0.43
Grade		
Elementary	100	1.35 ± 0.43
Middle	37	0.99 ± 0.32
Gender		
Male	68	1.33 ± 0.43
Female	69	1.19 ± 0.43
Family income (×10,000 won/month)^a^		
< 200	17	1.18 ± 0.38
200-399	47	1.20 ± 0.36
400-599	46	1.31 ± 0.46
> 600	23	1.25 ± 0.52
Level of parent’s education^a^		
≤ High school	61	1.29 ± 0.39
College/university	70	1.22 ± 0.48
Graduate school	5	1.22 ± 0.17
Father’s occupation		
Professional	17	1.18 ± 0.38
White collar/service	47	1.20 ± 0.36
Blue collar	46	1.31 ± 0.46
Unemployed	23	1.25 ± 0.52
Mother’s occupation		
Professional	24	1.31 ± 0.50
White collar/service	39	1.23 ± 0.42
Blue collar	11	1.21 ± 0.34
Unemployed	63	1.23 ± 0.40
Frequency of tooth cleaning		
≤ once a day	3	1.20 ± 0.12
2-3 times/day	101	1.29 ± 0.44
> 4 times/day	33	1.17 ± 0.40
Intake of sugar-containing sweets		
< Once a day	23	1.26 ± 0.40
Once a day	71	1.25 ± 0.44
2-3 times/day	43	1.27 ± 0.45
Dental treatment demand (1 year)		
Yes	80	1.27 ± 0.45
No	56	1.23 ± 0.41
History of decayed teeth		
Yes	63	1.31 ± 0.40
No	74	1.21 ± 0.46
CPI index		
0	34	1.19 ± 0.39
≥ 1	92	1.29 ± 0.44
Gingival index		
≤ 1	39	1.20 ± 0.41
2-3	98	1.28 ± 0.44
Plaque index		
≤ 2	56	1.15 ± 0.46
3	81	1.33 ± 0.40

*Abbreviations*: *Pb* lead

^a^Some answers were missing

Table 2 shows the distribution of demographic characteristics, socioeconomic status, oral health behavior, and dietary habits of the subjects after dividing into the BLL quartiles. Based on measured BLL, the study population was divided into equal quartile groups (I, II, III, and IV), with quartile I composed of subjects with the lowest BLLs. There were no statistically significant differences observed among the quartile groups.

**Table 2:** Distribution of demographic characteristics, socioeconomic status, oral health behavior, and dietary habits of children according to blood lead level quartiles

	Total (*n* = 137)	Quartile I (*n* = 35)	Quartile II (*n* = 34)	Quartile III (*n* = 34)	Quartile IV (*n* = 34)
	No. (%)	No. (%)	No. (%)	No. (%)	No. (%)
Age (years, mean ± SD)	10.9 ± 2.1	12.4 ± 1.7	11.1 ± 2.1	10.2 ± 1.9	9.8 ± 1.8
Gender					
Male	68 (49.6)	12 (34.3)	17 (50.0)	19 (55.9)	20 (58.8)
Female	69 (50.4)	23 (65.7)	17 (50.0)	15 (44.1)	14 (41.2)
Family income (10,000 won/month)^a^					
< 200	17 (12.8)	4 (11.4)	8 (24.2)	1 (2.9)	4 (12.9)
200-399	47 (35.3)	14 (40.0)	11 (33.3)	14 (41.2)	8 (25.8)
400-599	46 (34.6)	9 (25.7)	12 (36.4)	11 (32.4)	14 (45.2)
> 600	23 (17.3)	8 (22.9)	2 (6.1)	8 (23.5)	5 (16.1)
Parent’s education^a^					
≤ High school	61 (44.9)	11(31.4)	19 (55.9)	11 (33.3)	20 (58.8)
College/university	70 (51.5)	24 ()	13 (38.2)	19 (57.6)	14 (41.2)
Graduate school	5 (3.7)	–	2 (5.9)	3 (9.1)	–
Father’s occupation					
Profession	50 (36.5)	10 (28.6)	12 (35.3)	13 (38.2)	15 (44.1)
White collar/Service	45 (32.8)	15 (42.9)	8 (23.5)	10 (29.4)	12 (35.3)
Blue collar	34 (24.8)	8 (22.9)	11 (32.4)	10 (29.4)	5 (14.7)
Unemployed	8 (5.8)	2 (5.7)	3 (8.8)	1 (2.9)	2 (5.9)
Mother’s occupation					
Profession	24 (17.5)	6 (17.1)	3 (8.8)	10 (29.4)	5 (14.7)
White collar/Service	39 (28.5)	10 (28.6)	8 (23.5)	9 (26.5)	12 (35.3)
Blue collar	11 (8.0)	1 (2.9)	6 (17.6)	2 (5.9)	2 (5.9)
Unemployed	63 (46.0)	18 (51.4)	17(50.0)	13 (38.2)	15 (44.1)
Frequency of tooth cleaning					
≤ Once a day	3 (2.2)	–	1 (0.7)	2 (5.9)	–
2-3 times/day	101 (73.7)	25 (71.4)	24 (17.5)	27 (79.4)	25 (73.5)
> 4 times/day	33 (24.1)	10 (28.6)	9 (26.5)	5 (14.7)	9 (26.5)
Sugar-containing sweets intake					
< Once a day	23 (16.8)	5 (14.3)	5 (14.7)	7 (20.6)	6 (17.6)
Once a day	71 (51.8)	18 (51.4)	22 (64.7)	16 (47.1)	15 (44.1)
2-3 times/day	43 (31.4)	12 (34.3)	7 (20.6)	11 (32.4)	13 (38.2)
Dental treatment demand (1 year)					
Yes	80 (58.8)	20 (57.1)	21 (61.8)	17 (50.0)	22 (66.7)
No	56 (41.2)	15 (42.9)	13 (38.2)	17 (50.0)	11 (33.3)

^a^Some answers were missing

Distribution of oral health parameters according to the BLL quartiles is shown in Table 3. Participants with a higher BLL were more likely to have a high PI value (*p* < 0.015). There were no other statistically significant differences.

**Table 3:** Distribution of oral health parameters according to blood lead level quartiles

	Total (*n* = 137)	Quartile I (*n* = 35)	Quartile II (*n* = 34)	Quartile III (*n* = 34)	Quartile IV (*n* = 34)	*P*-value
	No. (%)	No. (%)	No. (%)	No. (%)	No. (%)	
Presence of dental caries						
DMFT (mean ± SD)	1.5 ± 1.9	1.4 ± 1.5	1.7 ± 2.3	1.1 ± 1.5	1.7 ± 2.1	0.477^a^
History of dental caries (DMFT ≥1)						
Yes	63 (46.0)	11 (31.4)	17 (50.0)	18 (52.9)	17 (50.0)	0.252^*^
No	74 (54.0)	24 (68.6)	17 (50.0)	16 (47.1)	17 (50.0)	
Community periodontal index						
mean ± SD	1.1 ± 0.8	0.9 ± 0.8	1.2 ± 0.7	1.2 ± 0.7	1.1 ± 0.8	0.358^a^
0	34 (27.8)	13 (41.9)	7 (23.3)	4 (12.1)	10 (31.3)	0.083
≥ 1	92 (73.0)	18 (58.1)	23 (76.7)	29 (87.9)	22 (68.8)	
Gingival index						
mean ± SD	2.1 ± 0.9	1.8 ± 0.9	2.1 ± 0.8	2.3 ± 0.7	2.0 ± 0.9	0.150^a^
≤ 1	37 (28.5)	15 (42.9)	8 (23.5)	5 (14.7)	11 (32.4)	*0.061* ^*^
2-3	98 (71.5)	20(57.1)	26 (76.5)	29 (85.3)	23 (67.6)	
Plaque index						
mean ± SD	2.6 ± 0.5	2.4 ± 0.5	2.6 ± 0.5	2.7 ± 0.4	2.7 ± 0.5	*0.015* ^a^
≤ 2	56 (40.9)	21 (60.0)	15 (44.1)	9 (26.5)	11(32.4)	*0.025* ^*^
3	81 (59.1)	14 (40.0)	19 (55.9)	25 (73.5)	23 (67.6)	

*Abbreviations*: *DMFT* decayed/missing/filled surfaces of permanent teeth, *CPI* community periodontal index, *GI* gingival index, *PI* plaque index

*p-value obtained from χ^2^-test

^a^Data analyzed by ANOVA

Italics are statistically significant (*p*<0.05)

Results of logistic regression analysis for the association between BLL and oral health parameters are shown in Table 4. A significant association was found in the third quartile between BLL and all oral health parameters. Subjects quartile III had a crude OR of 5.24 (95% CI: 1.48-18.56), 4.35 (95% CI: 1.36-13.9), and 4.17 (95% CI: 1.50-11.54) for CPI, GI, and PI, respectively, while those in the fourth quartile had a crude OR of 3.14 [95% CI: 1.14-8.41] for PI.

**Table 4:** Association between blood lead level (quartiles) and oral health parameters

		Model 1		Model 2		Model 3	
		OR	95%CI	OR	95%CI	OR	95%CI
CPI (outcome)							
Lead level	Quartile I	Ref.		Ref.		Ref.	
	Quartile II	2.37	0.79-7.18	3.14	0.93-10.55	*3.44*	*1.00-11.90*
	Quartile III	*5.24*	*1.48-18.56*	*7.66*	*1.84-31.91*	*7.21*	*1.72-30.19*
	Quartile IV	1.59	0.57-4.47	2.38	0.69-8.21	2.65	0.75-9.44
GI (outcome)							
Leadlevel	Quartile I	Ref.		Ref.		Ref.	
	Quartile II	2.44	0.86-6.88	*3.29*	*1.06-10.19*	*3.58*	*1.12-11.40*
	Quartile III	*4.35*	*1.36-1.90*	*6.80*	*1.80-25.68*	*6.13*	*1.62-23.19*
	Quartile IV	1.57	0.59-4.19	2.50	0.77-8.19	2.81	0.83-9.47
PI (outcome)							
Leadlevel	Quartile I	Ref.		Ref.		Ref.	
	Quartile II	1.90	0.73-4.95	1.66	0.61-4.52	1.60	0.58-.40
	Quartile III	*4.17*	*1.50-11.54*	*3.41*	*1.12-10.40*	*3.37*	*1.10-10.34*
	Quartile IV	*3.14*	*1.14-8.41*	2.47	0.80-7.60	2.37	0.76-7.38

Model 1: Unadjusted model

Model 2: Model adjusted for age and gender

Model 3: Model adjusted for age, gender, parent education level, and frequency of tooth brushing

*Abbreviations*: *CI* confidence interval, *CPI* community periodontal index, *GI* gingival index, *PI* plaque index, *OR* odds ratio, *Ref* reference quartile

Italics are statistically significant (*p*<0.05)

We constructed an age and gender adjusted model, which showed that subjects in the third quartile had an OR of 7.66 (95% CI: 1.84-31.91), 6.80 (95% CI: 1.80-25.68), and 3.41 (95% CI: 1.12-10.40) for CPI, GI, and PI, respectively, while those in the second quartile had an OR of 3.29 [95% CI: 1.06-10.19] for GI.

Finally, following adjustments for age, gender, parent education level, and frequency of tooth brushing, subjects in the third quartile had an OR of 7.21 (95% CI: 1.72-30.19), 6.13 (95% CI: 1.62-23.19), and 3.37 (95% CI: 1.10-10.34) for CPI, GI, and PI respectively, while those in the second quartile had an OR of 3.44 [95% CI: 1.00-11.90] and 3.58 [95% CI: 1.12-11.40] for CPI and GI, respectively.

## Discussion

The aim of the present study was to investigate the relationship between BLL and oral health in children. We recruited subjects from participants in the CHEER study, a national representative study performed in Korea that focused on the influence of living environment on the well-being of children. We found that higher BLLs were positively correlated with worse oral health measurements, including CPI, GI, and PI. Furthermore, in logistic regression analysis, that effect was maintained after adjusting for age, gender, parent education level, and frequency of tooth brushing.

The mean BLL of the children enrolled in this study was 1.25 ± 0.43 μg/dl and ranged from 0.36 to 2.90 μg/dl, which was lower than noted in previous studies conducted in China (5.56 μg/dL) and the United States (2.30 μg/dL) [19, 25]. However, even with this relatively low concentration of lead, we found evidence of its adverse effects on the oral health of our subjects.

Our results are consistent with those of previous studies of adults. According to a study conducted by El-Said et al. that focused on the risk of gingivitis in lead-exposed workers, gingivitis is related to lead sulfide, a product of the reaction between lead and hydrogen sulfide during food fermentation, which can produce gum irritation [26]. According to their study, the risk of gingivitis in exposed workers was 4.82 times higher than in non-exposed workers. Arora et al. also reported that cumulative lead exposure might increase the risk of tooth loss in adults [14].

Although the biological link between lead exposure and periodontal disease has not been clearly elucidated, an increase in reactive oxygen species (ROS) has been suggested to be a factor, as ROS are generated by lead exposure and known to cause oxidative stress [27]. Lee et al. reported an association between BLL and oxidative stress in adults [28]. ROS can also cause damage to proteins and DNA, as well as lipid peroxidation [29], and an increased level can lead to damage to gingival, periodontal ligament, and alveolar bone tissues [30]. Furthermore, lead in blood has been suggested to disturb the function of the salivary gland, which may contribute to plaque accumulation [31]. For example, heavy metals such as lead imitate calcium in some ways. Therefore lead may interfere with calcium metabolism which can alter normal cell function acutely and perturbation of calcium-metabolism may cause disorder of salivary gland function [32–34]. Available evidences showed that administration of lead resulted in 30-40% diminishment of salivary flow rates in rats [31]. However, there was no study to support this hypothesis in case of people. These factors might explain the results of our study.

The current study has some limitations. First, since the subjects of our study were recruited from only 2 cities in South Korea, the findings are not representative of the overall Korean population. In addition, since this is a cross-sectional study, the results must be cautiously interpreted because this is not causality but association study. As for another limitation, final sample size was not enough to have statistical power when including several confounders in logistic regression models resulting in non-significant association at the stratum of the fourth quartile. However, this result provides meaningful evidence that relatively higher blood lead in children was associated with poorer gingival health. Despite these limitations, to the best of our knowledge, this is the first epidemiologic study to present results regarding the effects of lead exposure on periodontal health in children in Korea.

## Conclusions

In conclusion, our findings indicate a relationship between BLL and oral health problems in children, especially gingivitis. Because of the adverse effects on oral health, attention must be paid even in cases with a low BLL and strategies devised to lower lead levels in children.

## Abbreviations

ANOVA: Analysis of variance
BLL: Blood lead level
CHEER: Children’s Health and Environment Research
CPI: Community periodontal index
DMFT: Decayed, missing, and filled surfaces of permanent teeth
GI: Gingival index
OR: Odds ratios
PI: Plaque index
WHO: World Health Organization
